# Supplementary material for: Spatiotemporal variation of malaria incidence in parasite clearance interventions and non-intervention areas in the Amhara Regional State, Ethiopia
Source: PLoS One. 2022 Sep 19;17(9):e0274500. doi: 10.1371/journal.pone.0274500 (PMC9484658; doi:10.1371/journal.pone.0274500)
Supplement: Attachment — Submitted filename: Response to Reviewers.docx [file pone.0274500.s003.docx]

**Author’s Response to Academic Editor’s and Reviewers’ Comments:**

**Manuscript No:** PONE-D-22-05813

**Title:** Spatiotemporal variation of malaria incidence in parasite clearance interventions and non-intervention areas in the Amhara Regional State, Ethiopia.

We would like to thank the academic editor and the reviewers for the thorough review of our manuscript, encouraging words, helpful comments, and the opportunity to resubmit a revised copy of the manuscript. We have addressed all the comments as suggested by the academic editor and reviewers in our manuscript, as indicated below:

**Response to Academic Editor’s Comments:**

**Comment**:

1. Please ensure that your manuscript meets PLOS ONE's style requirements, including those for file naming. The PLOS ONE style templates can be found at

<https://journals.plos.org/plosone/s/file?id=wjVg/PLOSOne_formatting_sample_main_body.pdf> and

<https://journals.plos.org/plosone/s/file?id=ba62/PLOSOne_formatting_sample_title_authors_affiliations.pdf>

**Response**:

The manuscript is revised using PLOS ONE’s style template found in the above links to meet the journal requirements, now the manuscript meets PLOS ONE’s style requirements (Please see the marked-up and unmarked copies of the revised documents).

**Comment:**

2. Please provide additional details regarding participant consent. In the ethics statement in the Methods and online submission information, please ensure that you have specified (1) whether consent was informed and (2) what type you obtained (for instance, written or verbal, and if verbal, how it was documented and witnessed). If your study included minors, state whether you obtained consent from parents or guardians. If the need for consent was waived by the ethics committee, please include this information.

If you are reporting a retrospective study of medical records or archived samples, please ensure that you have discussed whether all data were fully anonymized before you accessed them and/or whether the IRB or ethics committee waived the requirement for informed consent. If patients provided informed written consent to have data from their medical records used in research, please include this information.

**Response**:

The study was conducted using retrospective data which were collected using the DHIS2 reporting platform. The data were aggregated (no individual data was collected) and no individual identifiers were attached to the data. Now, the ethical consideration section of the manuscript is updated based on your comments.

**Comments**:

3. In your Data Availability statement, you have not specified where the minimal data set underlying the results described in your manuscript can be found. PLOS defines a study's minimal data set as the underlying data used to reach the conclusions drawn in the manuscript and any additional data required to replicate the reported study findings in their entirety. All PLOS journals require that the minimal data set be made fully available. For more information about our data policy, please see <http://journals.plos.org/plosone/s/data-availability>.

Upon re-submitting your revised manuscript, please upload your study’s minimal underlying data set as either Supporting Information files or to a stable, public repository and include the relevant URLs, DOIs, or accession numbers within your revised cover letter. For a list of acceptable repositories, please see <http://journals.plos.org/plosone/s/data-availability#loc-recommended-repositories>. Any potentially identifying patient information must be fully anonymized.

Important: If there are ethical or legal restrictions to sharing your data publicly, please explain these restrictions in detail. Please see our guidelines for more information on what we consider unacceptable restrictions to publicly sharing data: <http://journals.plos.org/plosone/s/data-availability#loc-unacceptable-data-access-restrictions>. Note that it is not acceptable for the authors to be the sole named individuals responsible for ensuring data access.

We will update your Data Availability statement to reflect the information you provide in your cover letter.

**Response**:

Due to third-party restrictions, data are available from the Amhara Public Health Institute (APHI). Interested researchers may contact the institute to get permission to access the data. You can contact the institute through email: [admin@aphi.gov.et](mailto:admin@aphi.gov.et) or [aphi172008@gmail.com](mailto:aphi172008@gmail.com); Office phone number: +251582263227. In this revised manuscript, a minimal dataset uploaded as a supporting file after getting the permission. The data availability section of the manuscript is now updated in response to the editor’s comments and mentioned in the cover letter.

**Comments**:

4. We note that Figure 1, 5 and 6 in your submission contain map images which may be copyrighted. All PLOS content is published under the Creative Commons Attribution License (CC BY 4.0), which means that the manuscript, images, and Supporting Information files will be freely available online, and any third party is permitted to access, download, copy, distribute, and use these materials in any way, even commercially, with proper attribution. For these reasons, we cannot publish previously copyrighted maps or satellite images created using proprietary data, such as Google software (Google Maps, Street View, and Earth). For more information, see our copyright guidelines: <http://journals.plos.org/plosone/s/licenses-and-copyright>.

We require you to either (1) present written permission from the copyright holder to publish these figures specifically under the CC BY 4.0 license, or (2) remove the figures from your submission:

 a. You may seek permission from the original copyright holder of Figure 1, 5 and 6 to publish the content specifically under the CC BY 4.0 license.

We recommend that you contact the original copyright holder with the Content Permission Form (<http://journals.plos.org/plosone/s/file?id=7c09/content-permission-form.pdf>) and the following text:

“I request permission for the open-access journal PLOS ONE to publish XXX under the Creative Commons Attribution License (CCAL) CC BY 4.0 (<http://creativecommons.org/licenses/by/4.0/>). Please be aware that this license allows unrestricted use and distribution, even commercially, by third parties. Please reply and provide explicit written permission to publish XXX under a CC BY license and complete the attached form.”

Please upload the completed Content Permission Form or other proof of granted permissions as an "Other" file with your submission.

 In the figure caption of the copyrighted figure, please include the following text: “Reprinted from [ref] under a CC BY license, with permission from [name of publisher], original copyright [original copyright year].”

 b. If you are unable to obtain permission from the original copyright holder to publish these figures under the CC BY 4.0 license or if the copyright holder’s requirements are incompatible with the CC BY 4.0 license, please either i) remove the figure or ii) supply a replacement figure that complies with the CC BY 4.0 license. Please check copyright information on all replacement figures and update the figure caption with source information. If applicable, please specify in the figure caption text when a figure is similar but not identical to the original image and is therefore for illustrative purposes only.

The following resources for replacing copyrighted map figures may be helpful:

USGS National Map Viewer (public domain): <http://viewer.nationalmap.gov/viewer/>

The Gateway to Astronaut Photography of Earth (public domain): <http://eol.jsc.nasa.gov/sseop/clickmap/>

Maps at the CIA (public domain): <https://www.cia.gov/library/publications/the-world-factbook/index.html> and <https://www.cia.gov/library/publications/cia-maps-publications/index.html>

NASA Earth Observatory (public domain): <http://earthobservatory.nasa.gov/>

Landsat: <http://landsat.visibleearth.nasa.gov/>

USGS EROS (Earth Resources Observatory and Science (EROS) Center) (public domain): [http://eros.usgs.gov/#](http://eros.usgs.gov/)

Natural Earth (public domain): <http://www.naturalearthdata.com/>

**Response**:

The issue regarding the copyright of the maps is valid and very important. I’m aware of that before using it. The maps are the official maps of the country and its lower-level structure. I used the maps to show the study areas and the result of the study (this is the only reason). The maps are published by the author, and the shapefiles I used to prepare the maps were the property of the Ethiopia Central Statistical Agency (CSA) after getting permission to use it. We acknowledge the CSA of Ethiopia for providing the shapefiles.

**Comments**:

5. Please include your tables as part of your main manuscript and remove the individual files. Please note that supplementary tables (should remain/ be uploaded) as separate "supporting information" files.

**Response**:

All the individual tables are now included in the main manuscript in response to the editor’s comments. Now there are no individual files in the revised submission. In case you want to get access to the individual files, you can find them in the first submission.

**Reviewers' comments:**

**Reviewer's Responses to Questions**

**Comments to the Author**

1. Is the manuscript technically sound, and do the data support the conclusions?

The manuscript must describe a technically sound piece of scientific research with data that supports the conclusions. Experiments must have been conducted rigorously, with appropriate controls, replication, and sample sizes. The conclusions must be drawn appropriately based on the data presented.

Reviewer #1: Partly

Reviewer #2: Yes

**Response**:

Now, the manuscript is revised in response to the comments, and the minimum dataset as supporting information is uploaded in this revised manuscript.

2. Has the statistical analysis been performed appropriately and rigorously?

Reviewer #1: Yes

Reviewer #2: Yes

**Response**: It is ok,

3. Have the authors made all data underlying the findings in their manuscript fully available?

The [PLOS Data policy](http://www.plosone.org/static/policies.action#sharing) requires authors to make all data underlying the findings described in their manuscript fully available without restriction, with rare exception (please refer to the Data Availability Statement in the manuscript PDF file). The data should be provided as part of the manuscript or its supporting information, or deposited to a public repository. For example, in addition to summary statistics, the data points behind means, medians and variance measures should be available. If there are restrictions on publicly sharing data—e.g. participant privacy or use of data from a third party—those must be specified.

Reviewer #1: No

Reviewer #2: Yes

**Response**: Due to third-party restrictions, data are available from the Amhara Public Health Institute (APHI). Interested researchers may contact the institute to get permission to access the data. You can contact them through email: [admin@aphi.gov.et](mailto:admin@aphi.gov.et) or [aphi172088@gmail.com](mailto:aphi172088@gmail.com); Office phone number: +251582263227. In this revised manuscript, a minimal dataset is uploaded as a supporting file after getting permission.

4. Is the manuscript presented in an intelligible fashion and written in standard English?

PLOS ONE does not copyedit accepted manuscripts, so the language in submitted articles must be clear, correct, and unambiguous. Any typographical or grammatical errors should be corrected at revision, so please note any specific errors here.

Reviewer #1: Yes

Reviewer #2: Yes

Response: It is ok.

5. Review Comments to the Author

Please use the space provided to explain your answers to the questions above. You may also include additional comments for the author, including concerns about dual publication, research ethics, or publication ethics. (Please upload your review as an attachment if it exceeds 20,000 characters)

Reviewer #1: I suggest that the article be rewritten, adding explanations about the geographical organization of the country, its administrative division. This understanding is necessary to be able to assess the selection of study regions.
Futhermore it was not very clear what interventions had been carried out. In addition, to having selected a small number of kebele in relation to the total studied.

**Response**: In collaboration with the government and partners, malaria parasite clearance interventions (mass testing and treatment followed by focal testing and treatment) were implemented in six kebeles (the lowest administrative unit in Ethiopia) of eight districts of Amhara Regional State, Ethiopia (mentioned in the introduction section). The study map showed the geographic organization of the country (Fig 1). The intervention kebeles were selected purposively (Scott et al. Malar J).

**Comment**:

Reviewer #2: The article has relevance and originality for publication by the journal, but needs adjustments for a better presentation:

The title is relevant, concise and aligned with the general objective of the work.

The abstract demonstrates that the research is relevant and has been constructed logically.

Suitable keywords

Introduction is contextualized, updated and presented in an objective and coherent way, leading the reader to the objective of the study.

Methodology was appropriate and satisfactory for the purpose of the study.

The results are presented and discussed in depth, and it is possible to qualify the study with the addition of illustrative figures and tables, facilitating the reading and analysis of the data. Note regarding the positioning of Figure 2 and Figure 3 in the work, where Figure 3 is before Figure 2.

Discussion is coherent and sufficient for a good understanding of the study. Punctual suggestions should be made by the authors in order to better qualify the article, such as changing the positioning of lines 338, 339 and 340 for Conclusion.

The authors conclude based on the results, but similarly to the discussion, it is recommended to remove lines 348, 349 and 350 from the Conclusion and insert them in discussion.

Up-to-date and consistent bibliography. All authors cited in the text were described in the reference.

My opinion is PARTIALLY SATISFACTORY recommending this study for publication in PLOS ONE, as soon as the authors comply with the comments indicated in the text.

**Response**: Regarding the position of figures, (Fig 2) is about the seasonal decomposition of malaria incidence in the intervention kebeles whereas (Fig 3) is about the seasonal decomposition of malaria incidence in the non-intervention kebeles. We can change the position of the figures if it is logical. Lines 338, 339, and 340 of the discussion are removed and inserted in the conclusion section of the manuscript. Lines 348, 349, and 350 are rephrased and kept in the original place to conclude the spatiotemporal distribution of malaria. If we removed and inserted the paragraph in the discussion section, the manuscript lacks a conclusion regarding the spatiotemporal distribution of malaria. Now, comments are considered, and the manuscript is updated in response to the reviewers’ comments.

**Thank you all for your comments!**
